# Gaucher: A Systematic Review on Oral and Radiological Aspects

**DOI:** 10.3390/medicina59040670

**Published:** 2023-03-28

**Authors:** Giuseppe Minervini, Rocco Franco, Maria Maddalena Marrapodi, Vini Mehta, Luca Fiorillo, Almir Badnjević, Gabriele Cervino, Marco Cicciù

**Affiliations:** 1 Multidisciplinary Department of Medical-Surgical and Odontostomatological Specialties, University of Campania “Luigi Vanvitelli”, 80121 Naples, Italy; 2 Department of Biomedicine and Prevention, University of Rome “Tor Vergata”, 00100 Rome, Italy; 3 Department of Woman, Child and General and Specialist Surgery, University of Campania “Luigi Vanvitelli”, 80100 Naples, Italy; 4 Department of Public health Dentistry, Dr. D. Y. Patil Dental College and Hospital, Dr. D. Y. Patil Vidhyapeeth University, Pune 411014, India; 5 Department of Biomedical and Dental Sciences and Morphofunctional Imaging, School of Dentistry, University of Messina, 98125 Messina, Italy; 6 Verlab Research Institute for Biomedical Engineering, Medical Devices and Artificial Intelligence, 71000 Sarajevo, Bosnia and Herzegovina; 7 Department of General Surgery and Medical-Surgical Specialties, School of Dentistry, University of Catania, 95131 Catania, Italy

**Keywords:** Gaucher disease, bone, oral health, congenital disorders

## Abstract

*Background and Objectives*: Gaucher disease (GD) is a lysosomal storage disorder with the genetic autosomal recessive transmission. Bone involvement is a prevalent finding in Gaucher disease. It causes deformity and limits daily activities and the quality of life. In 75% of patients, there is bone involvement. This review aims to evaluate the principal findings in the jaw by a Cone-beam computed tomography (CBTC) and X-ray orthopantomography; *Materials and Methods*: PubMed, Web of Science, Lilacs and Scopus were systematically searched until 31 December 2022. In addition, a manual search was performed using the bibliography of selected articles and a Google Scholar search. Clinical studies were selected that considered principal radiographic findings in radiography in a group of patients affected by GD. *Results*: Out of 5079 papers, four studies were included. The main findings are generalized rarefaction and enlarged narrow space, anodontia. *Conclusions*: The exact mechanism of bone manifestation is probably due to the infiltration of Gaucher cells in the bone marrow and, consequently, the destruction of bone architecture. All long bones are a potential means of skeletal manifestation. The jaw is more affected than the maxilla, and the principal features are cortical thinning, osteosclerosis, pseudocystic lesions, mental demineralization, flattening in the head of the condyle, effacement of anatomical structures, thickening of maxillary sinus mucosa. The dentist plays a crucial role in diagnosing and treating these patients. Sometimes the diagnosis can be made by a simple panoramic radiograph. All long bones are affected, and the mandible is particularly involved.

## 1. Introduction

Gaucher disease (GD) is a lysosomal storage disorder with genetic autosomal recessive transmission [1,2,3,4]. The mutation of the β-glucocerebrosidase gene causes the malfunction of the lysosomal enzyme glucocerebrosidase. Cells, especially macrophages, that undergo glucocerebrosidase accumulation are called Gaucher cells. These cells, called Gaucher cells, become dilated and have a cytoplasm with an engorged, wrinkled tissue paper appearance and are displaced around the nuclei [5,6,7]. GD has an incidence of 1 in 50,000 to 100,000 people in the general population. Still, there is an increase among communities with consanguineous marriages, inbreeding, or geographically limited groups with an expected birth rate of 1:850 among the Ashkenazi Jewish population [8]. In some geographical areas, such as the Norrbottnian region of Northern Sweden, there is a higher incidence of GD with a particular form of the disease [1,9]. The main features of the disorder are due to the infiltration of Gaucher cells into the principal organs of the reticuloendothelial system, such as spleen, liver, and bone marrow [10,11]. The mutated gene is located on chromosome 1q22 and is inherited paternally. It is formed by ten introns and 11 exons [12,13,14,15]. Nowadays, 300 mutations are discovered as the cause of GD [16]. N370S, L444P, 84GG, and IVS2 are several gene loci most frequently involved in mutation for the onset of GD, with a prevalence of 98% [17,18]. The first two influence clinical manifestations, as some others several genetic diseases [13,14]. GD causes significant morbidity and disability; in types 1 and 3 many organs of the skeletal system are involved, while in type two the visceral and neurological blood system are involved so as in others many several oro-craniofacial diseases. [19,20,21,22]. The skeletal manifestations include osteopenia, pathological fractures, growth retardation, osteoporosis, focal lytic or sclerotic lesions, bone pain, painful or bone crisis, decreased mineralization [23,24,25,26], osteonecrosis or vascular necrosis, cortical and medullary infarcts [21,27,28]. Anemia and thrombocytopenia are the early signs of the most common hematologic manifestations [7,9,11,15]. The infiltration of engorged macrophages in the spleen, liver, and bone marrow causes a depression of hematopoiesis, leading to thrombocytopenia [11,29]. Other hematologic manifestations include monoclonal and polyclonal gammopathies, which are risk factors for neoplasms as multiple myeloma [7,30,31]. The most affected organs in Gaucher disease are the liver and spleen, which increased in volume due to macrophage accumulation in Kupffer cells [11]. Nevertheless, portal hypertension is rare due to cirrhosis and fibrosis [4]. Spleen volume is normally 5–15 times greater in type 1; however, it can sometimes significantly increase and exceed 50 times normal. Massive splenomegaly may cause fibrosis and increase the risk of rupture and malignancies. In type 1 GD, the most frequent neurological manifestation is Parkinson’s disease, while in type 2 and 3, central nervous system (CNS) manifestations, including dementia and epilepsy, are more frequent, so a multidisciplinary approach it is necessary [7,13,21,30,32]. Recently, a new manifestation of myoclonic epilepsy has been connected to Gaucher’s disease. Bleeding is an important sign and manifests itself as frequent epistaxis, easy bruising, and hemorrhaging after surgical/or dental procedures or during pregnancy or childbirth, so that the clinician must be prepared in the management of these various possible unexpected events in the different fields of medicine and dentistry [9,13,33,34,35,36,37]. The abnormal bleeding is caused by hypersplenism and the infiltration of bone marrow by Gaucher cells. GD is associated with some abnormal platelet function or malfunction of clotting [10]. The diagnosis is made through the measurement of low levels of enzyme activity in peripheral blood cells. Sometimes, molecular genotype analyses are important to evaluate the possible evolution of the disease [27]. There is no cure for Gaucher’s disease; in 1991, intravenous infusions of enzyme replacement therapy (ERT) were approved. However, it only treats symptomatic episodes, while asymptomatic episodes are untreated. Early ERT improves hepatosplenomegaly, hematologic manifestations, bone pain, and bone mineral density [10,11,38]. The symptomatic Gaucher Disease commonly involves the bones. The bone manifestation causes pain, difficulty in motility, and skeletal abnormalities, and it is a very limiting factor for the life of the individual, a differential diagnosis with other diseases must to be carefully obtained, thanks to the use of technologies and specific diagnostics methods [9,21,39]. The epidemiologic study of Germain estimated that 75% of patients with type 1 Gaucher Disease have a bone manifestation of the disease. With the improvement of radiologic and diagnostic techniques [40,41], 90% of patients have one or more bone manifestations [8,21]. The exact mechanism of bone manifestation is probably due to the infiltration of Gaucher cells in the bone marrow and, consequently, the destruction of bone architecture [7]. All long bones are a potential means of skeletal manifestation [21]. All long bones, including the mandible, are potential infiltration sites [28]. In the literature, about 100 cases describe the infiltration of the maxillo-mandibular complex noted on radiographs. The most common finding is the presence of radiolucent honeycomb areas in the premolar-molar region. The most common radiographic observation in an affected mandible is the presence of radiolucent pseudocystic or honeycomb lesions, mainly in the premolar-molar regions. There is also a loss of normal bone trabeculae [9]. Other findings include generalized osteoporosis, widening, and widening of bone marrow spaces, endosseous scallops and, in some cases, apical root resorption, all presumably due to Gaucher cell density in the apical regions. Cortical bone, however, remains intact. It has been hypothesized that the sclerotic areas are not empty, and this process is completely reversible [34,40]. In regard to the jaw, it is a possible focus on Gaucher cells infiltration [21]. In the literature, only 100 cases with jaw manifestations have been documented. The discovery is often accidental during a dental or panoramic X-ray [13,16]. The study aims to identify the principal bone jaw features involved in GD. This is a review that evaluates jawbone manifestations, which helps the dentist to make an early diagnosis.

## 2. Materials and Methods

### 2.1. Eligibility Criteria

All documents were assessed for eligibility based on the following population (including animal species), Exposure, Comparator, and Outcomes (PECO) [42]:

(P) Participants consisted of patients.

(E) The exposure consists of patients with GD and bone manifestations.

(C) The comparison was healthy patients with no GD history and other bone-related systemic diseases.

(O) The result is to evaluate the frequency and incidence of bone lesions detected by radiology in GD patients compared with healthy patients. The secondary purpose is to assess the differences in oral health (caries index and periodontal disease) between the GD and healthy patient groups.

Only papers providing data at the end of the intervention were included. Exclusion criteria were the following: (1) Studies on GD with no radiographic exams; (2) Studies with groups of patients suffering from other systemic diseases; (3) deals with bone manifestations in other anatomical districts; (4) cross-over study design; (5) studies written in a language different from English; (6) full-text unavailability (i.e., posters and conference abstracts); (7) studies involving animals; (8) review articles; (9) case reports.

### 2.2. Search Strategy

The study used the main scientific databases (PUBMED, WEB of SCIENCE, LILACS, SCOPUS). The time window considered for the electronic search was from 1 March 1990 to 31 December 2022. The term “Gaucher disease” was first combined with “bone” and then independently with “oral health” using the Boolean connector “OR”. The web search was assisted using MESH (Medical Subjects Headings) (Table 1). The keywords used in the search engine using MeSh are as follows: (“Gaucher disease” [MeSH Terms] OR (“gaucher” [All Fields] AND “disease” [All Fields] OR “gaucher disease” [All Fields] AND (“bone and bones” [MeSH Terms] OR (“bone” [All Fields] AND “bones” [All Fields] OR “bone and bones” [All Fields] OR “bone” [All Fields] OR (“oral health” [MeSH Terms] OR (“oral” [All Fields] AND “health” [All Fields] OR “oral health” [All Fields]). In addition, a manual search was performed using the bibliography of found articles and a free search on Google Scholar.

This systematic review was conducted according to Preferred Reporting Items for Systematic Reviews (PRISMA) guidelines and the Cochrane Handbook for Systematic Reviews of Interventions. The systematic review protocol was registered on the International Prospective Register of Systematic Reviews (PROSPERO) with the following number CRD42022333235 on 21 April 2022.

### 2.3. Data Extraction

Two reviewers (GM and RF) independently extracted data from the included studies using a customized data extraction on a Microsoft Excel sheet. In disagreement, a consensus was reached through a third reviewer (MC).

The following data were extracted: (1) First author; (2) Year of publication; (3) Nationality; (4) Age of study participants; (5) Sample; (6) Radiographic signs; (7) Evaluation of oral health.

### 2.4. Quality Assessment

The risk of bias in papers was assessed by two reviewers using Version 2 of the Cochrane risk-of-bias tool for randomized trials (RoB 2) (Cochrane Corp., Fredericksburg, VA, USA). Any disagreement was discussed until a consensus was reached with a third reviewer.

## 3. Results

### 3.1. Study Characteristics

After searching the three search motors, 5079 articles were selected. The exclusion criteria automatically removed the review and non-English articles via the Boolean operator NOT. Specifically, 25 articles from LILACS, 261 from Web of Science, 273 from PubMed, and 350 from Scopus were deleted. A fourth search engine on Scopus was used, given the specificity of the topic. In addition, 1226 articles were eliminated as duplicates. During the first screening phase, 2953 articles were considered; however, according to the inclusion criteria, clinical trials and randomized controlled trials were considered, and so 2902 articles were excluded. One article was excluded because the full text could not be found.

**Figure 1:**
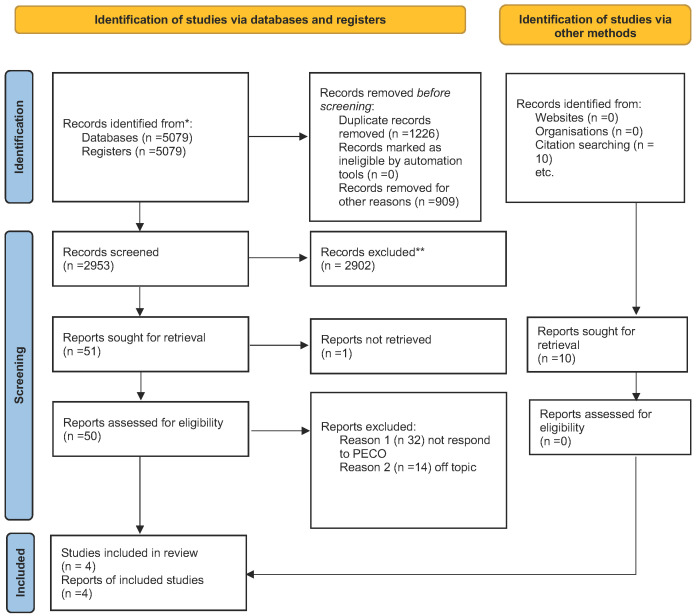
Prisma Flowchart. * papers identified by search methods; ** papers removed because systematic reviews of the literature.

Therefore, 50 articles were after this screening stage; the abstracts were read to assess eligibility. According to the PRISMA 2020 flowchart in Figure 1, only four were chosen for this review. The articles were excluded because they were either off-topic and did not meet PECO or were systematic literature reviews. Figure 1 shows the screening process and why articles were excluded from this systematic review. A total of 46 articles were excluded: 32 were eliminated because do not answer the question posed in Section 2 by PECO and therefore were included in this review (assessing the frequency of bone lesions in patients with GD and evaluating their oral health), and 14 were off-topic. According to the PECO model, four papers were chosen for title and abstract screening. The included studies have been published over the past 20 years (1983 to 2022). In parallel, a manual bibliography search of the selected articles and a search of the main sites were performed. From this it emerged that ten papers were selected. However, six were excluded because they were off-topic, and the remaining four articles coincided with those found in the databases. The studies analyzed were conducted in various parts of the world: South America (Brazil) and Israel. A total of 430 subjects with GD were analyzed. Regarding the study designs, there were four clinical studies. Among these four studies, three included a control group; all used DMFT to evaluate caries and Gingival Index (GI) to assess periodontal status. All studies evaluated radiographic evidence in the oral cavity by either orthopanoramic or Tc Cone Beam. Table 2 summarizes the main characteristics of all the study included in the present systematic review.

### 3.2. Main Findings

The study of Nobre et al. analyses the principal bone abnormalities of 10 GP. The study comprises a group of 10 patients affected by GD (4 males; 6 females) and a control group of 20 healthy patients. The patients underwent radiographic analysis (Cone Beam Tc and orthopantomography). All patients underwent an anamnestic control, intra- and extra-oral examination, and a CBCT and panoramic radiography. Although there was radiological evidence of bone involvement in all ten patients, only four had pathological fractures or delays in tooth eruption. During CBCT analysis, the jaw showed pathological features in all ten patients and the maxilla in six. The radiographic analysis revealed the presence of generalized rarefaction and enlarged marrow spaces in all patients. Other radiographic signs were cortical thinning, osteosclerosis (five patients), pseudocystic lesions (nine patients), mental demineralization (seven patients), flattening in the head of the condyle (one patient), effacement of anatomical structures (eight patients), thickening of maxillary sinus mucosa (three patients). The orthopantomography revealed signs in the mandible and in 8 maxillae. Afterwards, the author compared the radiographic findings in CBCT and orthopantomography with the study and control groups against a Fisher’s exact test. CBCT has more predictability to evaluate the following signs: generalized bone rarefaction (*p* = 0.0001) and TMJ involvement (*p* = 0.0002). CBCT is not an important tool to reveal other bone signs with statistical relevance. CBCT is more effective in highlighting differences between GD and control groups, thus proving an essential tool for evaluating patients with GD [15]. The second study by Mohamed et al. focuses on jaw involvement and radiographic features. The case-control study evaluates a panel of 42 GP (26 males and 16 females with an average age of 9.54 ± 4.25 years) and a control group of 84 (45 males and 39 females with a mean age of 11.37 ± 1.83 years). The patients all had Gaucher type 1 and type 3. The following features in the radiographic images were examined: generalized bone rarefaction, localized rarefaction and enlarged bone marrow spaces thinning of the cortex, pseudocystic radiolucent lesions, anodontia and dental anomalies. Cyst-like radiolucent lesions were defined as a pseudocyst. The biopsy was not performed due to the lack of symptoms of the lesions. Generalized rarefaction is a radiographic finding in type I and type 3. GD type III presents a localized rarefaction, but type I widens the bone marrow. The following signs are more frequent in type III: pseudocysts radiolucent lesions, cortex thinning, anodontia, and dental anomalies. Chi-squared test showed an association between types I and III and generalized rarefaction, wide bone marrow spaces, pseudocyst radiolucency, cortex thinning, dental abnormalities, and absence of abnormal radiographic features with a *p*-value < 0.05. Generalized rarefaction, wide bone marrow spaces, and cortex thinning are more frequent in type I GD, but pseudocysts are not associated with type I. The radiological features are not essential signs in type I (95% CI 0.03–0.39, *p*-value = 0.0009). On the other hand, type III is associated with some radiological features (generalized rarefaction, pseudocysts radiolucent lesions, thinning of the cortex, and dental anomalies). The widening of bone marrow is not a radiological feature of type III (*p*-value = 0.3464). In conclusion, the radiological features are associated with type III (odds ratio of 0.13, 95% CI 0.05–0.37, *p*-value = 0.0001) [21]. Fischman’s study analyzed a cohort of 350 patients who underwent a periodontal examination and radiological analysis. After the statistical analysis, the control patients showed a worse periodontal health status than those with this pathology. Affected patients showed better DMFS levels than carriers (36.8 vs. 49.4), with a *p* = 0.048. The most significant difference was found between MS (missing surfaces). Affected patients showed a halving of the missing surfaces, 9.5 versus 18.9, with a *p*-value of 0.008. The DMFS index between the two categories did not show large statistically significant differences. Therefore, this study showed no significant differences between periodontal health [18]. The study of Carter analyses 25 patients, and 25 of the 28 patients showed radiographic evidence of bone resorption. The most common finding is the enlargement of the medullary spaces. The most common result is the gross enlargement of the medullary spaces and the radiolucency and displacement of the mandibular canal. It has also been shown that delayed eruption of permanent teeth is present. Therefore, the alterations at the bone level are very significant and very frequent (Table 2) [17].

### 3.3. Quality Assessment and Risk of Bias

**Figure 2:**
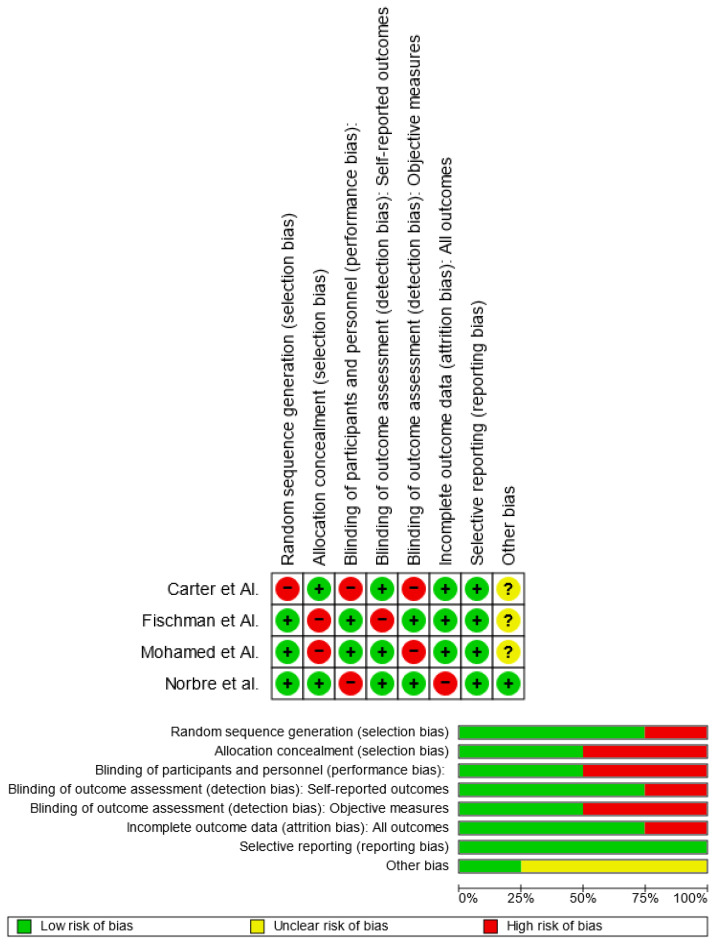
Risk-of-bias domains of included studies.

Using RoB 2, the risk of bias was estimated and reported in Figure 2. Regarding the randomization process, 75% of the studies ensured a low risk of bias. However, 50% of the studies excluded a performance bias, but 75% reported all outcome data, 50% of the included studies adequately excluded bias in the selection of reported outcomes, and 75% excluded bias in self-reported outcomes. Overall, all four studies were shown to have a low risk of experiencing bias.

## 4. Discussion

The involvement of bones is a prevalent finding in Gaucher disease. It causes deformity and limits daily activities and patients’ quality of life. In 75% of patients, there is bone involvement. Recent advances in diagnostic and imaging modalities have revealed that 90% of patients with type I or III Gaucher disease have one or more bone manifestations. Orthopedic prostheses are the only solution to replace the necrosis of bone and lytic changes [13,17,18,30,43]. The exact mechanism of bone manifestations is still uncertain, but the infiltration of Gaucher cells in the bone marrow is the most important feature [34,40]. All long bones are possible Gaucher cell targets. The mandible is classified as a long bone and is, therefore, involved. About 100 cases with maxilla-facial involvement are described and documented in the literature database. This is occasionally discovered during a radiographic survey [11,29]. Regarding oral symptoms, GD is frequently asymptomatic, but clinical examinations and regular radiographic exams can detect the disease’s early warning signs. Spontaneous gingival bleeding, yellowish skin pigmentation, petechiae on the oral mucosa, [11,15,16] and delayed tooth eruption are some of the most typical oral symptoms. In young GD patients, Fischman et al. 7 found a significant correlation between the delayed eruption of the permanent teeth and mild to severe bone involvement.

Asymptomatic mandibular bone disorders are common. The lamina dura has thinned, there is pervasive osteopenia with loss of trabecular bone structure, and the mandibular canal has been displaced by pseudocysts lesions, among other recorded radiographic abnormalities of this area. It has also been shown that nearby teeth, mostly molars and premolars, undergo apical root resorption. The maxilla is less frequently impacted when it primarily comprises resorption in the maxillary sinus region. The possible mechanism of the bone lesion and the presence of radiolucent areas are osteosclerotic reactions or abnormal bone regeneration in the post-extraction area. Bender et al. [7] assert that a dental panoramic X-ray is essential for diagnosis. The region between premolars is rich in bone marrow. Therefore, the presence of 12 cases with radiographic signs in the premolar region indicates that jawbone marrow is infiltrated by Gaucher cells [7,8,21]. Some cases have later shown a possible apices reabsorption without pulpal necrosis. The accumulation of Gaucher cells causes a scalloped appearance in the endosteal bone region [7,11,29,41]. The mandible and the maxilla manifest diffuse osteoporosis like the radiographic signs of other conditions such as thalassemia major and sickle cell anemia. Dental X-rays can often provisionally detect GD [9,15,21]. In the soft tissue, there are no important signs. In some cases, platelet dysfunction, oral pigmentation, or petechiae are to be interpreted as clinical findings, as highlighted in the study of Givol et al. [3,30], which evaluates the risk of bleeding after oral surgery in GD patients. Givol treated a group of GD patients undergoing hematologic analysis and a platelet function exam [30]. The study showed the following results: patients with Gaucher disease who suffer from platelet dysfunction must be treated by performing an accurate hemostasis. Platelet transfusions are recommended if there is a high risk of bleeding. The first study showed that the main features of GD generalized rarefaction in the CBCT are enlarged marrow, cortical thinning, osteosclerosis, pseudocysts lesion, and dental demineralization in seven. This study confirms that the mandible is more affected than the maxilla [15]. The second study analyzed the prevalence of radiographic features in the different types of GD [21,44]. Generalized rarefaction has a similar incidence in two kinds of GD; localized rarefaction is a clinical finding of type III, and widening of the bone marrow spaces is a clinical finding of type I [8,21]. Pseudocysts radiolucent lesions, cortex thinning, anodontia and dental anomalies are clinical findings in type III [21]. According to the previous study by Bender, Saranjam et al. [7,45], the above features are mainly found in the mandible due to the infiltration of GD cells in the marrow. According to Bender et al. [10] and Michanowiz et al. [46], the common radiological features in the premolar-molar region are the presence of pseudocystic or honeycombed radiolucent lesions. Bone manipulation creates a bone turnover and improves the radiolucent lesions [34,40]. The jaw is more affected than the maxilla. The delayed eruption of the teeth is widespread in GD, except if amyloidosis and other pathology take over [17]. GD is a risk factor for mucosal disease such as amyloidosis; the literature described only five cases, according to Elstein et al. [41,44]. The salivary flow is lower compared to the control. Spontaneous or surgically induced bleeding is widespread due to thrombocytopenia and the alteration of the coagulation cascade. According to the DMFS index, the patient’s dental health was equal to the controls. Compared to the control group, the patients had roughly half as many carious surfaces and half as many missing surfaces. Given that the carriers and the patients come from the same households, it is reasonable to presume that their socioeconomic circumstances and access to oral healthcare are similar. The negligible variation in filled surfaces, a measure of dental therapy, supports this notion. Though the patients may have had better health awareness, including greater concern for their dental health, they were aware of their Gaucher disease status. The observed differences between the DS and MS scores could be attributed to a healthier diet and improved dental hygiene. There was expected to be a connection between Gaucher disease and gingival disease because both conditions are characterized by anemia, a propensity to bleed, and poor healing. The patients may have adhered to better oral hygiene practices because they were aware of their “at risk” status, as was previously suggested. ERT helps the patient to control bleeding against the increase in platelets. In conclusion, the presence of a lesion, especially in the jaw, is a constant feature in dental radiography [7,30,47]. The dentist and must intercept this lesion to obtain a diagnostic suspicion and diagnose the disease. Today, thanks to new technologies that allow early diagnosis, it is possible to start therapy early in order to be able to reduce the adverse effects of the disease [48,49,50,51]. In this study, we analyzed the main radiographic features present radiographically in GD patients and oral health in this type of patient. Statistics showed that GD patients have radiographic manifestations that allow early diagnosis. Furthermore, the only study by Fischman et al. evaluated oral health and stated no statistically significant differences in periodontal and carious health. The limitations of the studies are that there has not been a classification and a study comparing the radiographic differences among the three subtypes and also a study analyzing whether any of these three subtypes have worse oral health. This is mainly due to the condition’s rarity, which does not allow for a statistically significant sample. Patients with Gaucher disease frequently report excruciating pain in different skeletal regions but rarely in the jawbones or craniofacial region. With 13 and 60 years of follow-up, Bender and Bender reported two instances of Gaucher disease; in the first case, mandibular lesions were present in the premolar-molar region, and the affected teeth were essential. These results matched the description of our patient. Additionally, Bender and Bender found that ERT improved the mandibular rarefaction bilaterally without showing any signs of osteolysis. However, according to some research, orthopedic intervention such as joint (hip, knee, or shoulder replacement) replacement is advised because ERT cannot reverse the necrotic and lytic changes in long bones. Additionally, our patient had a history of having a hip joint replaced and long bones affected by Gaucher disease. Bender and Bender claimed that without clinical and laboratory testing and in light of radiographic findings, it is impossible to make a conclusive diagnosis of Gaucher disease involving the jawbone without biopsy; however, other studies have only recommended biopsy in situations where other conditions are suspected in the differential diagnosis, such as in the case described here.

## 5. Conclusions

This review analyzed Gaucher’s disease’s primary clinical and radiological signs and symptoms. Although a rare pathology, all radiological and clinical signs must direct the dental specialist to a correct diagnosis. In addition, this study showed no variation in the oral health of patients with GD. Therefore, the dentist’s role is to establish and maintain a healthy periodontium and teeth. In addition, sometimes the dentist’s role in the early diagnosis of the disease may be necessary.

## Figures and Tables

**Table 1:** Search strategy.

***PubMed*** (gaucher disease) AND ((bone) OR (oral health))
***Web of Science *** TITLE-ABS-KEY (gaucher disease) AND ((bone) OR (oral health))
***Lilacs*** (gaucher disease) AND ((bone) OR (oral health))
***Scopus*** *TITLE-ABS-K*EY (((gaucher AND disease) AND ((bone) OR (oral AND health))))

**Table 2:** Main characteristics of the studies included in the present systematic review.

Authors	Year	Sample	Age	Radiographic Signs	Evaluation of Oral Health	Nationality
Norbre et al.	2012	10 with GD compared with 20 healthy	23.2 years	Generalized rarefaction and enlarged narrow space	No difference	Brazil
Mohamed et al.	2020	42 with GD compared with 84 healthy	11.37 years	Generalized rarefaction, pseudocysts radiolucent lesion, anodontia	No difference	Egypt
Fischman et al.	2003	350 with GD and 31 control	30.7 years	Bone involvement is frequent. The finding in the ortho-panoramic is always frequent	No statistical significance between DMFT, Gingival Index	Israel
Carter et al.	1998	28 with GD	32.4 years	The most common findings are enlargement of medullary spaces	No dental findings in oral health	Israel

## Data Availability

Not applicable.

## Abbreviations

GD	Gaucher disease
CNS	Central nervous system
ERT	Enzyme replacement therapy
PECO	Patients, Exposure, Comparison, Outcome
MeSH	Medical subjects headings
PRISMA	Preferred Reporting Items for Systematic Reviews and Meta-Analyses
GI	Gingival index
DMFT	Decayed, missing and filled teeth
CBCT	Cone beam tc
TMJ	Temporomandibular joint
MS	Missing surface
DMFS	Decayed, missing and filled surface
